# Integument Mycobiota of Wild European Hedgehogs (*Erinaceus europaeus*) from Catalonia, Spain

**DOI:** 10.5402/2012/659754

**Published:** 2012-09-25

**Authors:** R. A. Molina-López, C. Adelantado, E. L. Arosemena, E. Obón, L. Darwich, M. A. Calvo

**Affiliations:** ^1^Centre de Fauna Salvatge de Torreferrussa, Catalan Wildlife Service, Forestal Catalana, 08130 Santa Perpètua de la Mogoda, Spain; ^2^Departament de Sanitat i d'Anatomia Animals, Facultat de Veterinaria, Universitat Autònoma de Barcelona, 08193 Bellaterra, Barcelona, Spain; ^3^Centre de Recerca en Sanitat Animal (CReSA), UAB-IRTA, Campus de la Universitat Autònoma de Barcelona, 08193 Bellaterra, Barcelona, Spain

## Abstract

There are some reports about the risk of manipulating wild hedgehogs since they can be reservoirs of potential zoonotic agents like dermatophytes. The aim of this study was to describe the integument mycobiota, with special attention to dermatophytes of wild European hedgehogs. Samples from spines and fur were cultured separately in Sabouraud dextrose agar (SDA) with antibiotic and dermatophyte test medium (DTM) plates. Nineteen different fungal genera were isolated from 91 cultures of 102 hedgehogs. The most prevalent genera were *Cladosporium* (79.1%), *Penicillium* (74.7%), *Alternaria* (64.8%), and *Rhizopus* (63.7%). A lower prevalence of *Aspergillus* (*P* = 0,035; *χ* ^2^ = 8,633) and *Arthrinium* (*P* = 0,043; *χ* ^2^ = 8,173) was isolated during the spring time and higher frequencies of *Fusarium* (*P* = 0,015; *χ* ^2^ = 10,533) during the autumn. The prevalence of *Acremonium* was significantly higher in young animals (70%, 26/37) than in adults (30%, 11/37) (*P* = 0,019; *χ* ^2^ = 5,915). Moreover, the majority of the saprophytic species that grew at the SDA culture were also detected at the DTM. Finally, no cases of ringworm were diagnosed and no dermatophytes spp. were isolated. Concluding, this study provides the first description of fungal mycobiota of the integument of wild European hedgehogs in Spain, showing a large number of saprophytic species and the absence of dermatophytes.

## 1. Introduction

The European hedgehog (*Erinaceus europaeus*) is one of the two species of hedgehogs living in Catalonia (North-eastern Spain). In the area of study, this species is frequent in deciduous or semideciduous forests, in holm-oak woods and also in human-related habitats such as gardens, parks, and fields. Due to their distribution and behaviour, many hedgehogs are found in the wild and brought to wildlife rehabilitation centres in Europe [1, 2]. Several potential zoonotic agents have been reported in hedgehogs, mostly bacteria such as *Salmonella* spp. [3, 4], tick-borne diseases, [5, 6] and also fungi [7]. *Trichophyton mentagrophytes var. erinacei* is the most common causative agent of ringworm in wild hedgehogs [8] and 25% of carriers have been documented in a survey from England [9].

The skin is the largest mammalian organ and represents a complex microbial ecosystem with bacteria, viruses, protozoa, and fungi in direct contact with the environment. The potential association of the alterations of the skin mycobiota with disease has been well established in human medicine [10]. *Trichophyton mentagrophytes var. erinacei* has been isolated in zoophilic dermatophytoses in humans [11, 12] and an outbreak of ringworm in caretakers of wild European hedgehogs was reported in Germany [13].

There are several studies of mycobiota carried out with samples from domestic animals, like cats [14] and dogs [15]. In all these studies, the most frequently isolated fungal genera were classified as saprophytes (mainly *Aspergillus, Alternaria, Penicillium,* and *Cladosporium* spp.) but in cats two pathogenic genera (*Microsporum* and *Trichophyton*) have been also described.

Since there is scarce information about the mycobiota in wild hedgehogs, the aim of this study was to describe this mycobiota, with special attention to dermatophytes, in wild hedgehogs admitted at a wildlife rehabilitation centre in Catalonia (Spain).

## 2. Material and Methods

### 2.1. Animals

All wild European hedgehogs admitted at the Wildlife Rehabilitation Centre of Torreferrussa (Catalonia, north-east Spain, 3°19′-0°9′ E and 42°51′-40°31′ N) from June 2009 to August 2010 were included in the study. Sample collection was performed during the routinely clinical examination of hedgehogs at admission. Animals were anesthetized with isoflurane (Forane, Abbott) after chamber or mask induction, and one to three spines and hair were removed with a sterile mosquito forceps. Two sets of spines were collected from both the clavicular and the lumbar region. Hair samples were obtained from the ventral part of the body. The forceps were sterilized by flaming immediately before the sampling of each body part. 

The centre directly depends on the governmental Catalan Wildlife Service. Thus, protocols, amendments, and other resources were done according to the guidelines approved by the government of Catalonia.

### 2.2. Microbiological Analysis

Samples were cultured in Sabouraud dextrose agar (SDA) with antibiotics and dermatophyte test medium (DTM) plates. Spines and hair were cultured in separated plates. For the culture of spines, each plate was divided in two parts, one from the dorsal spine and the other from the lumbar one.

Samples were inoculated on duplicate plates of SDA (Difco) with chloramphenicol (400 ppm) and dermatophyte selective medium prepared according to Taplin's DTM formula (Biolife) [16].

The plates were incubated at 28°C and regularly examined for a month. Taxonomic identification of all the different mycelial colonies was based on macroscopic and microscopic studies. The observation was made from fragments of the colony prepared freshly by adding blue lactophenol. The strains were observed under light microscope. According to the fungal structures observed, identification of strains at genus level was performed. The identification of yeasts was performed by staining with methylene blue and using the API 20C system.

### 2.3. Statistical Analysis

The following data were recorded: age, sex, date of admission, cause of hospitalization, main diagnosis, and location where hedgehogs were found. Age was categorized as juveniles or adults, according to their body weight and the date of admission. Data analysis was carried out using SPSS version 15.0 statistical software (Chicago, Illinois, USA). Data were summarized with standard descriptive statistics. Prevalence and its 95% confidence intervals (95% CIs) were calculated. Differences were evaluated by the *χ* ^2^ test for qualitative variables, and their 95% CIs were calculated. Kruskall-Wallis test with the percentiles 25% and 75% was applied as a nonparametric test.

## 3. Results

A total of 102 hedgehogs were examined during the period of the study. Results were obtained from 91 cultures. The causes of admission and demographic characteristics of these animals are summarized in Table 1. No cases of ringworm were diagnosed and no dermatophyte isolation was obtained in any of the cultures. Moreover, the majority of the saprophytic species that grew on SDA medium were also detected at the DTM. In some strains of the genera *Cladosporium*, *Penicillium, *and *Aspergillus* a colour change was also detected at the DTM.

The prevalence of the different fungal genera isolated from the wild hedgehog is summarized in Table 2. Nineteen different genera were finally isolated. The most prevalent genus were *Cladosporium* (79.1%), *Penicillium* (74.7%), *Alternaria* (64.8%), and* Rhizopus* (63.7%). No significant differences were observed in the prevalence of genera among the different anatomical regions (cranial and caudal spines or fur). Only two genus, *Rhizopus* and *Trichoderma* were more frequently found in spines than in fur and 4 species *(Aphanocladium *spp.*, A. ochraceus, Phoma *spp., and* Ulocladium* spp.) were only isolated from the fur (Table 2).

As regards the season of the year, we found lower prevalence of *Aspergillus* (*P* = 0,035; *χ* ^2^ = 8,633) and *Arthrinium* (*P* = 0,043; *χ* ^2^ = 8,173) in the spring and higher frequencies of *Fusarium* (*P* = 0,015; *χ* ^2^ = 10,533) in the autumn.

The number of species isolated from the different body areas was similar: cranial (median = 3; P_25_ = 2; P_75_ = 5); caudal (median = 3; P_25_ = 2; P_75_ = 5), and fur (median = 3; P_25_ = 1; P_75_ = 4). Moreover, no statistical differences were observed between prevalence of fungal species and the cause of admission, the clinical signs, or the sex. By contrast, the prevalence of *Acremonium* was significantly higher in young animals (70%, 26/37) than in adults (30%, 11/37) (*P* = 0,019; *χ* ^2^ = 5,915).

## 4. Discussion

This work provides the first description of the normal mycobiota of the integument of wild European hedgehog in a region of Spain. Interestingly, a large variety of saprophytic fungi were isolated in these animals, although no dermatophytes were found.

Hedgehogs are one of the most common wild mammals attended at the different rehabilitation centres in Europe, due to their abundance and their vicinity to humans. Moreover, the reduced body size and relatively tameness of these animals are factors that favour their capture and handling. Ringworm caused by *T. mentagrophytes var. erinacei* has been reported in wild European hedgehogs, clinically characterized by crusty lesions, alopecia, and loss of spines, mainly affecting the head area [17]. In Britain, Morris and English [9] reported a prevalence of 19.8% (39/203) of *T. mentagrophytes var. erinacei* in hair samples in a survey which included both alive and dead animals. In their study, many of infected cases carried the fungus without lesions. Thus, the potential role of wild European hedgehogs as a source of human infection and its public health significance have been emphasized in different reports [18, 19]. Different species of dermatophytes have been commonly reported in healthy mammals with different prevalence levels depending on the animal species; for instance, 4% of cats [20], 8.1% of dogs [21], 8.8% of goats, and 12.2% of sheep [22] were positive to dermatophytes. By contrast, in some animal species, such as in dromedary camels [23], it has not been possible to find dermatophytes.

The majority of the fungal species isolated in this work are ubiquitous and most of the genera match with those reported in human toes and in the hair of other mammalian species. In the toes of humans, at least fourteen different genera of fungi have been isolated [24]. These genera included yeasts such as *Candida albicans*, *Rhodotorula rubra*, *Torulopsis glabra, *and *Trichosporon cutaneum*; dermatophytes such as *Microsporum gypseum* and *Trichophyton rubrum;* opportunistic fungi that can live in skin such as *Rhizopus stolonifer*, *Trichosporon cutaneum*, *Fusarium *spp, *Scopulariopsis brevicaulis*, *Curvularia *spp, *Alternaria alternata*, *Paecilomyces *spp, *Aspergillus flavus,* and *Penicillium* spp. Other authors reported *T. mentagrophytes* and *T. rubrum* as the most common dermatophytes in a study carried out in 360 human patients [25]. Among nondermatophytes the order of decreased incidence was *Scopulariopsis brevicaulis*, *Acremonium roseum*, *Aspergillus* spp, and *Fusarium* spp. On the other hand, frequencies of saprobe genera higher than 50% have been described in dogs of the same geographical area (Catalonia) of our study by Cabañes et al. 1996 [21], and also in cats from Brazil [16] and Iran [20]. These saprophytic species are frequently found in the environment, soil, or plants. Moreover, *Chaetomium* and *Trichoderma* are known for their cellulolytic activity, so their presence may be related to the natural environment in which the wild hedgehogs live.

There are evidences in the literature that pet [26, 27] and wild [13] hedgehogs can be the source of pathogenic fungal infections in humans. The absence of *Trichophyton sp*. in hedgehogs of this study was unexpected, but some facts could contribute to this outcome. All examined animals were wild hedgehogs sampled the first day of the admission and no clinical signs indicative of ringworm were observed in any of the animals; likely, they had no time to suffer immunodepression due to the capture-associated stress and, in consequence, no time for spreading any subclinical infection in case of wild reservoirs. Moreover, the total number of saprophytic fungal genera isolated in this work was higher than those published by others [19, 28]. The presence of saprophytic species has been considered as an indicator of transient integument contamination from soil or environment [28]. This high prevalence of saprophyte mycobiota could interfere with the growth of other pathogenic species, like dermatophytes if they are in a lower proportion. More sensitive techniques, like PCR, could help in determine this role in wild reservoirs. Nonetheless, in the present study, the mycotic culture was conducted in two different mediums (DTM and SDA), and in three different anatomical locations, corroborating the absence of these pathogens in all the cultures.

The following genera: *Acremonium, Alternaria, Aspergillus, Mucor, Rhizopus, Absidia, Alternaria, Fusarium,* and *Penicillium,* detected in integument of the hedgehogs of our study, have been reported as opportunistic pathogens in immunocompromised humans [29] and animals [28], causing cutaneous or systemic mycoses. However, those genera are considered ubiquitous and the potential role of the hedgehogs as carriers or reservoirs could be considered as negligible. Although no cases of mycoses have been observed in our study population, the potential risk in primary or secondary immunodepressed hedgehogs should be considered. *Neosartorya hiratsukae*, an ascomycete in which the conidial state resembles *Aspergillus fumigatus, *was described in a captive exotic African pygmy hedgehog [30]. This pathogen is known to cause human fatal brain infection [31].

In conclusion, our study provides the first description of the integument mycobiota of wild European hedgehogs in Spain, showing a large number of saprophyte species in both spines and fur and the absence of dermatophytes. Further larger studies are necessary to elucidate the relevancy of the seasonal and age group differences observed.

## Figures and Tables

**Table 1:** Descriptive data of the causes of admission and demographic characteristics of the 91 wild European hedgehogs examined.

	Number (%)			
Cause	Young		Adult	
	Male	Female	Male	Female
Incidental^a^	19 (21)	14 (15.4)	9 (9.9)	16 (17.6)
Trauma	5 (5.5)	4 (4.4)	6 (6.6)	1 (1)
Weakness/natural disease	4 (4.4)	4 (4.4)	1 (1)	8 (8.8)

^a^This category also includes abandoned young healthy animals.

**Table 2:** Prevalence of fungal genera and species isolated from different regions of wild hedgehogs.

	Prevalence (%)						
Mycobiota genus/specie	Spines		*P* value	Fur	*P* value		Overall
	Cranial area (Cr)	Caudal area (Cd)	Cr-Cd	ventral	Fur-Cr	Fur-Cd	prevalence
*Absidia*	2.2	3.3	ns	1.1	ns	ns	3.3
*Acremonium*	24.2	26.4	ns	20.9	ns	ns	40.7
*Alternaria*	47.3	36.3	**0.013**	39.6	ns	ns	64.8
*Arthrinium*	28.6	27.5	ns	35.2	ns	ns	42.9
*Aphanocladium*	0.0	0.0	—	1.1	—	—	1.1
*Aspergillus * spp.	38.5	37.4	ns	34.1	ns	ns	54.9
*A. * *ochraceus *	0.0	0.0	—	1.1	—	—	1.1
*A. * *flavus *	5.5	5.5	ns	5.5	ns	ns	8.8
*A. * *niger *	11.0	11.0	ns	5.5	ns	ns	17.6
*Aureobasidium*	1.1	3.3	ns	4.4	ns	ns	7.7
*Chaetomium*	4.4	5.5	ns	6.6	ns	ns	9.9
*Cladosporium*	59.3	52.7	ns	51.6	ns	ns	79.1
*Epicoccum*	1.1	0.0	—	0.0	—	—	1.1
*Fusarium*	11.0	8.8	ns	15.4	ns	ns	23.1
*Mucor*	11.0	14.3	ns	5.5	ns	**0.039**	17.6
*Penicillium*	59.3	48.4	ns	51.6	ns	ns	74.7
*Phoma*	0.0	0.0	—	1.1	—	—	1.1
*Rhizopus*	51.6	49.5	ns	28.6	**0.001**	**0.02**	63.7
*Rhodotorula*	1.1	3.3	ns	0.0	—	—	4.4
*Saccharomyces*	1.1	0.0	—	2.2	ns	—	3.3
*Trichoderma*	20.9	20.9	ns	7.7	**0.002**	**0.002**	24.2
*Ulocladium*	0.0	0.0	—	2.2	—	—	2.2

Ns: statistically not significant (*P* > 0.05).
